# On force generation in electro-fluidic linear actuators with ferrofluid

**DOI:** 10.1038/s41598-022-26190-2

**Published:** 2022-12-24

**Authors:** Matthew O. T. Cole, James Moran

**Affiliations:** Department of Mechanical Engineering, Chiang Mai University, Chiang Mai, 50200 Thailand

**Keywords:** Mechanical engineering, Materials science

## Abstract

In ferrofluid actuation systems, forces are generated by actively controlling pressure and flow within the fluid using an applied magnetic field. There are multiple contributing factors in force generation involving complex non-linear couplings between electromagnetic and fluid pressure fields. This brings significant challenges in theory-based design and optimization. In this paper, a theoretical model of pressure transmission between a ferrofluid and solid is derived starting from Maxwell’s stress tensor and accounting for magnetic saturation within the fluid. This model shows that linear actuator designs based on orthogonal mode operation, where the field direction through the fluid is perpendicular to the motion direction, can provide the highest force capacity for a given field strength from the actuator coil. This is verified by theoretical analysis of some basic linear actuator topologies. The results are applied in the design and analysis of a novel piston-type linear actuator with sealed chamber and two internal electrical coils for bidirectional operation. Experimental measurements of both static and dynamic behaviour are shown to validate the described principles. The actuator produces smooth and precise flow-regulated motion, has zero intrinsic stiffness, and exhibits very low friction due to the suspension effect from ferrofluid layers within the actuator.

## Introduction

Ferrofluid is a type of smart magnetic fluid containing a suspension of magnetically polarized nano-particles, typically of iron oxide or iron-cobalt alloy^1,2^. The suspended particles are coated with a surfactant to prevent aggregation and sedimentation. This renders the pressure and flow within a ferrofluid controllable by an applied magnetic field. Over the past few decades, ferrofluids have found wide-ranging applications within the fields of science, medicine and engineering^3–7^.

Ferrofluids possess high magnetic permeability, thermal conductivity, and viscosity, when compared with air and other types of fluid^8–10^. Consequently, they can be used to enhance the performance of conventional electromagnetic actuation systems, including Lorentz force (voice coil) actuators^11,12^. Ferrofluids can also provide a fundamentally different method of actuation where motion of a mechanical system is dependent on pressure and flow within the fluid, controlled directly via an electromagnetic field^13–15^. Various applications for ferrofluid actuators in high precision and micro-scale motion control systems have been proposed^4,14–17^. Presently, there are significant challenges in creating compact ferrofluid actuators for large displacement range and force capacity, as desired in many micro-positioning systems. The work described here addresses these challenges by developing and applying the theory of force generation with ferrofluids in the context of linear actuation systems. Case studies are presented for designs based on two different modes of operation, where the magnetic field through the fluid is parallel and orthogonal to the direction of motion/actuation. These results lead to a novel design of a bidirectional ferrofluid actuator that is manufactured and studied experimentally. Theoretical predictions of both static and dynamic behaviour are compared with the experimental results to validate the theory and design principles.

Although this study is aimed to facilitate the optimal design of ferrofluidic linear actuation systems, the results are relevant to other situations where functional pressure is generated through a ferrofluid, and the resulting combination of fluid and magnetic pressures must be predicted and analysed. These include ferrofluid bearings, vibration isolators and dampers, valves, pumps, as well as other emerging applications of force and motion control with ferrofluids.

The mechanism by which ferrofluids can produce buoyancy forces on immersed objects with low magnetic permeability has been studied extensively since ferrofluids were first created in the 1960’s^18,19^. The principle of force generation in these situations is that any displacement causing a localized increase in magnetic flux density will result in a corresponding increase in fluid pressure in that region, thereby producing a restoring force that will tend to stabilize the supported object’s position. The same principle can be exploited in the creation of fluid-film bearings that combine electrical or permanent magnets with ferrofluid^1,20–23^. For some newly proposed ferrofluid actuation systems, permanent magnets have been used to generate magnetic fields that are then varied in strength differentially using electrical coils^6,16^. In such cases, the permanent magnet field produces conservative restoring forces that do not contribute to the net energy transfer of the actuator, yet introduce an intrinsic stiffness to the actuator that limits the displacement range (stroke length).

From the basic actuator topologies considered in the present work, an optimal mode of force generation is determined where the magnetic field is perpendicular to the actuation axis so that magnetic pressure forces are less affected by actuator displacement and by magnetic saturation within the ferrofluid. The results show the significant potential of ferrofluid actuators to combine a number of unique features and advantages, including:

- Smooth and precise flow-regulated motion of an actuator having no intrinsic stiffness.
- Larger force capacity and/or stroke length compared with similarly sized conventional electromagnetic actuators.
- Active force control is combined with passive damping properties from the fluid flow, where both properties can be designed for separately.
- Bearing suspension properties of ferrofluids can be exploited to eliminate solid-to-solid contact and thereby avoid non-viscous stick-slip friction effects.

A number of drawbacks and challenges still exist, and these are reflected in the paper’s conclusions.

## Analysis of force generation with ferrofluids

For a solid body in contact with a magnetic fluid, the resultant force depends on the hydraulic pressure from the fluid in summation with the magnetic field stress at the solid-fluid interface. The isotropic pressure in a ferrofluid is a superposition of the magnetic pressure, which arises due to magnetization, and the underlying hydraulic pressure that would occur also if the fluid was nonmagnetic fluid. A unique feature of magnetic fluid systems is their capacity to generate both positive and negative pressure on a solid object, depending on the direction of the applied field and the magnetic properties of the solid. However, how the presence of ferrofluid affects the field, magnetic pressure and resulting fluid pressure is difficult to predict by simple calculation, and this makes the design of ferrofluid actuation systems challenging.

### Governing equations

A general theory for electromagnetic actuation with ferrofluids can be derived from the stress tensor for force transmission within a magnetized fluid. This is a modified version of the Maxwell stress tensor that takes into account the hydraulic pressure within the fluid^24^:

1 $$\begin{aligned} {\textbf{T}}=-{\textbf{I}}(p^{*}+\frac{\mu _{0}}{2}H^{2})+\varvec{H}\varvec{B}^{T} \end{aligned}$$

Here, $\varvec{I}$ is the $3\times 3$ identity matrix, $\varvec{H}$ is the magnetic field vector (with magnitude denoted by *H*), $\varvec{B}$ is the induced flux vector and $\mu _{0}$ is the permeability of free space. For an incompressible isothermal ferrofluid, the isotropic pressure $p^{*}$ at a point within the fluid is dependent on the field magnitude at that point, according to

2 $$\begin{aligned} p^{*}=p+\mu _{0}\int M.dH \end{aligned}$$

where *p* is the residual nonmagnetic pressure. Magnetic hysteresis is very low for most common ferrofluids, so the magnetization *M* may be treated as a single-valued function of the applied field *H*. Accordingly, the magnetization pressure component in Eq. (2) is

3 $$\begin{aligned} p_{m}(H)=\mu _{0}\int _{0}^{H}M(H).dH \end{aligned}$$

A generalized Bernoulli equation can also be defined for an incompressible and inviscid magnetic fluid, and can be applied along any streamline in steady-state flows^24^:

4 $$\begin{aligned} p^{*}-p_{m}+\rho gh+\tfrac{1}{2}\rho v^{2}=\textrm{constant} \end{aligned}$$

**Figure 1:**
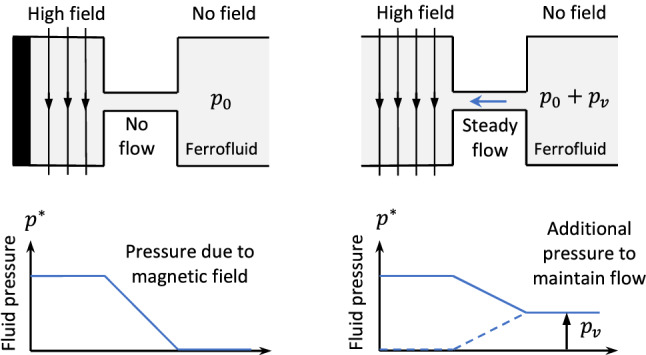
Illustration of pressure components due to applied magnetic field, both with and without fluid flow.

This equation is a statement of conservation of energy, where $p^{*}$ accounts for the work done by changes in fluid pressure, $p_{m}$ corresponds to the work done by magnetic forces and the final two terms correspond to gravitational and kinetic energy respectively. For hydrostatic problems we can write

5 $$\begin{aligned} p^{*}-p_{m}+\rho gh=\textrm{constant}=p_{0} \end{aligned}$$

where $p_{0}$ is the pressure at a point in the fluid where the height is zero ($h=0$) and the field is zero (so that the magnetization pressure is also zero). This equation implies that, without gravity, the underlying nonmagnetic pressure $p=p^*-p_m$ is constant throughout the fluid—not the actual fluid pressure $p^{*}$, as would be the case for nonmagnetic fluids. A further remarkable consequence is that a ferrofluid can flow from low pressure regions to high pressure regions if the pressure difference is generated by an applied magnetic field. This situation is illustrated in Fig. 1.

**Figure 2:**
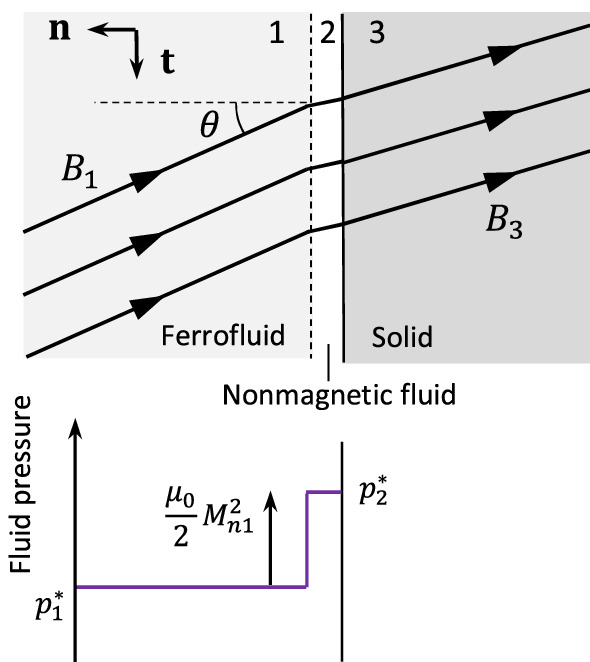
Magnetic field and fluid pressure near an interface with a magnetic fluid.

For analysis purposes, the magnetization behaviour of a uniform colloidal ferrofluid can be reasonably described by the nonlinear Langevin function:

6 $$\begin{aligned} M(H)=M_{s}\left( \coth \frac{H}{H_{0}}-\frac{H_{0}}{H}\right) \end{aligned}$$

where the saturation magnetization $M_{s}$ and saturation field strength $H_{0}$ depend on the properties and concentration of the suspended particles. This function can be integrated analytically to obtain the magnetization pressure:

7 $$\begin{aligned} p_m(H)=\mu _0 M_{s}H_{0}\left[ \ln \left( \sinh \frac{H}{H_{0}}\right) -\ln \frac{H}{H_{0}}\right] \end{aligned}$$

A limitation of this model is its neglect of particle-particle interactions, which can cause underestimation of the low-field susceptibility, especially for high particle concentrations^8^.

### Force transmission

To analyze and understand the force transmission mechanism, we may consider an interface between a target object made of solid material (medium 3) and a ferrofluid (medium 1) subjected to a uniform magnetic field oriented with angle $\theta$ to the normal, as shown in Fig. 2. In general, the force due to contact with a ferrofluid is not equal to the isotropic pressure within the fluid due to the additional traction effect of the magnetic field on the fluid. To show this, it is useful to conceptualize a thin layer of nonmagnetic fluid (medium 2) that separates the ferrofluid and solid and transmits a contact force via the fluid pressure only.

The pressure acting on the solid object is equal to the stress transmitted through the ferrofluid in the direction of the normal vector $\varvec{n}$. This can be evaluated from the stress tensor according to $p_{act}=-\varvec{n}^{T}{\textbf{T}}_{1}\varvec{n}$. From Eq. (1),

8 $$\begin{aligned} \varvec{n}^{T}{\textbf{T}}_{1}\varvec{n}=-(p_{1}^{*}+\tfrac{1}{2}\mu _{0}H_{1}^{2})+\varvec{n}^{T}\varvec{H}_{1}\varvec{B}_{1}^{T}\varvec{n} \end{aligned}$$

Given that $\varvec{B}_{1}=\mu _{0}(\varvec{H}_{1}+\varvec{M}_{1})$, this may be expressed

9 $$\begin{aligned} \varvec{n}^{T}{\textbf{T}}_{1}\varvec{n}=-p_{1}^{*}-\tfrac{1}{2}\mu _{0}H_{1}^{2}+\mu _{0}H_{n1}^{2}+\mu _{0}H_{n1}M_{n1} \end{aligned}$$

where $H_{n1}=\varvec{n}^{T}\varvec{H}_{1}=H_{1}\cos \theta$ denotes the normal component of the field. Equation (1) can also be applied to the nonmagnetic fluid layer 2, giving

10 $$\begin{aligned} \varvec{n}^{T}{\textbf{T}}_{2}\varvec{n}=-p_{2}^{*}-\tfrac{1}{2}\mu _{0}H_{2}^{2}+\mu _{0}H_{n2}^{2} \end{aligned}$$

The fields in media 1 and 2 are related by the standard continuity conditions:

11 $$\begin{aligned} B_{n2}=B_{n1},\,\,H_{t2}=H_{t1} \end{aligned}$$

where $H_{t}=\varvec{t}^{T}\varvec{H}$ denotes the tangential component of $\varvec{H}$. Therefore, from Eq. (11),

12 $$\begin{aligned} H_{n2}&=\frac{1}{\mu _{0}}B_{n2}=\frac{1}{\mu _{0}}B_{n1}=H_{n1}+M_{n1}\end{aligned}$$

13 $$\begin{aligned} H_{2}^{2}&=H_{n2}^{2}+H_{t2}^{2}=H_{1}^{2}+2M_{n1}H_{n1}+M_{n1}^{2} \end{aligned}$$

Substituting Eqs. (12) and (13) in Eq. (10) gives

14 $$\begin{aligned} \varvec{n}^{T}{\textbf{T}}_{2}\varvec{n}=-p_{2}^{*}+\tfrac{1}{2}\mu _{0}M_{n1}^{2}-\tfrac{1}{2}\mu _{0}H_{1}^{2}+\mu _{0}H_{n1}^{2}+\mu _{0}H_{n1}M_{n1} \end{aligned}$$

The normal stress must be continuous at the boundary, implying $\varvec{n}^{T}{\textbf{T}}_{1}\varvec{n}=\varvec{n}^{T}{\textbf{T}}_{2}\varvec{n}$. Therefore, equating Eqs. (9) and (14), we obtain

15 $$\begin{aligned} p_{2}^{*}=p_{1}^{*}+\tfrac{1}{2}\mu _{0}M_{n1}^{2} \end{aligned}$$

As medium 2 is nonmagnetic, $p_{2}^{*}$ given by Eq. (15) is the mechanical contact pressure acting on medium 3. This differs from the isotropic pressure within the ferrofluid $p_{1}^{*}$ wherever the normal magnetization component $M_{n1}$ is nonzero. Suppose that, away from the interface, there is a point in the fluid where the pressure takes a known value $p_{0}$, and where the magnetic field is negligible. In this case, according to Eq. (5), we have $p_{1}^{*}=p_{0}+p_{m}$ and so the contact pressure is given by

16 $$\begin{aligned} p_{2}^{*}=p_{0}+p_m(H_{1})+\tfrac{1}{2}\mu _{0}M_{n1}^{2} \end{aligned}$$

This equation may be used to calculate the resultant force on a nonmagnetic object, as the magnetic field through the target object then gives no contribution to the resultant force.

In general, the total actuation pressure will be a summation of the contact pressure and magnetic field pressure. The total pressure (above ambient $p_0$) transmitted to the solid object follows from Eq. (9) as

17 $$\begin{aligned} p_{act}=p_m(H_1) + \mu _{0}\left( \tfrac{1}{2}H_{1}^{2}-H_{n1}^{2}-H_{n1}M_{n1}\right) \end{aligned}$$

For a ferrofluid with linear magnetization properties, the relation $\varvec{M}=\chi \varvec{H}$ can be applied, where the susceptibility $\chi$ is a constant. In this case, $p_m(H)=\mu _0\frac{1}{2}\chi H^{2}$ and Eq. (17) simplifies to

18 $$\begin{aligned} p_{act}=\mu _{0}(\chi +1)\left( \tfrac{1}{2}-\cos ^{2}\theta \right) H_{1}^{2} \end{aligned}$$

**Figure 3:**
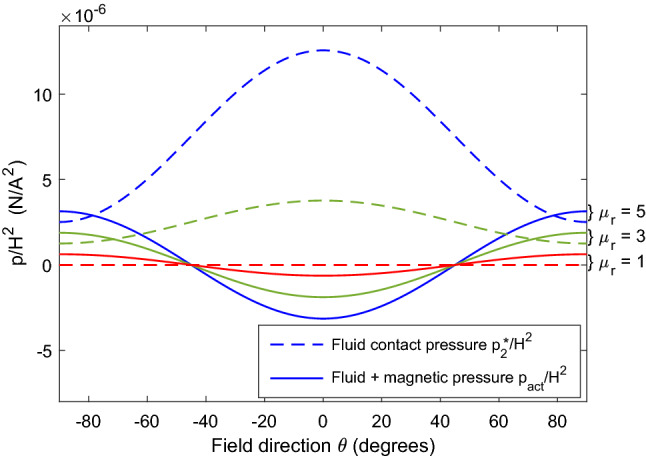
Dependency of interface pressures on magnetic field direction (angle of incidence) for ferrofluids with different linear magnetization properties.

The preceding analysis shows how both the isotropic fluid pressure and magnetic forces contribute to the total transmitted pressure. The graph in Fig. 3 shows the quantity $p_{act}/H_{1}^{2}$ (having dimensions of force per current squared) as a function of field direction. The total pressure magnitude is equally large for $\theta =0^{\circ }$ and $\theta =\pm 90^{\circ }$. However, if the field is parallel to the actuation direction ($\theta =0^{\circ }$), there is a magnetic traction that is partly cancelled by the fluid pressure, whereas if the field is orthogonal to the axis ($\theta =\pm 90^{\circ }$), the fluid pressure and magnetic pressure combine constructively. As these curves are based on linear magnetization properties, the transmitted force increases in proportion to the fluid’s relative permeability $\mu _{r}=\chi +1$ for any given field direction.

These results point to the design of actuation systems based on two different modes of operation: with the field either parallel or orthogonal to the actuation axis. In both cases, to what extent the interface pressure can be exploited for useful work will depend on the overall design and geometry of the actuator. For a real physical system, the behaviour will differ from the idealized situation in the following ways:

1. The resultant force acting on a target object of finite dimensions will depend on the magnetic field over its entire surface, so the field should be directed through the body to maximize the force in the direction of motion.
2. There will be a variation in field direction over the surface of the target object (e.g. due to flux leakage/divergence) so that the idealized case of uniform unidirectional flux is unachievable in practice.
3. For large field strengths, the magnetization of the ferrofluid will saturate, causing a reduction in magnetization pressure compared with the idealized linear case.

All three effects can diminish the capacity of an actuator to do useful work and are therefore key considerations in design practice. For the first two items, general analysis is difficult as the results will depend greatly on the topology and geometry of the actuator. Instead, these issues are investigated through numerical and experimental case studies described in later sections. Item 3 is readily accounted for within the previously described theory and is discussed further in the following subsection.

**Figure 4:**
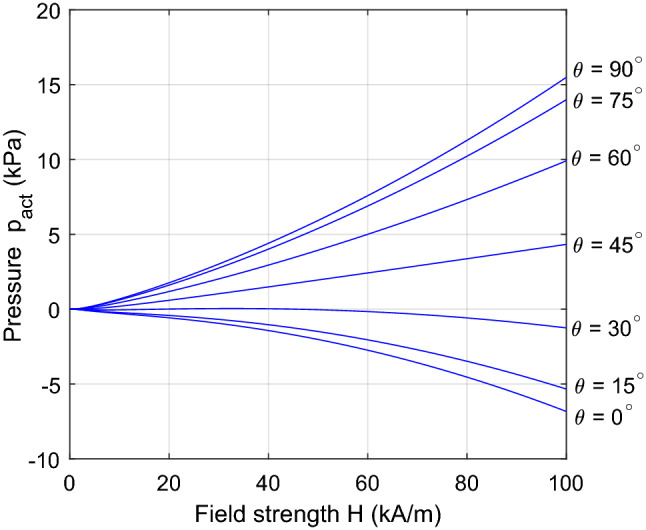
Dependency of transmitted pressure $p_{act}$ on field strength for different field directions $\theta$ when magnetization saturation is accounted for.

**Figure 5:**
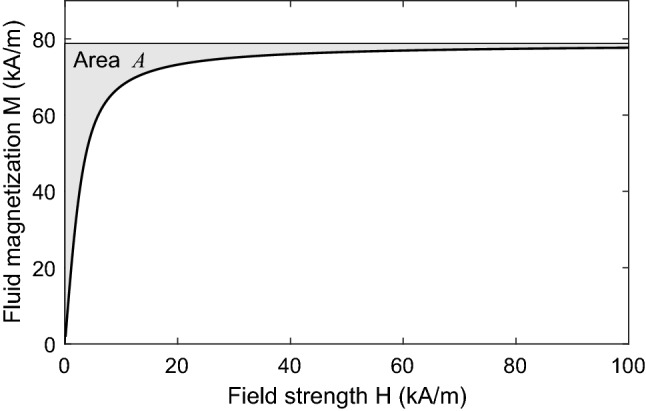
Ferrofluid magnetization curve, based on Langevin function with $M_{s}=79$ $\mathrm {kA/m}$ and $H_{0}=1.41$ kA/m.

**Figure 6:**
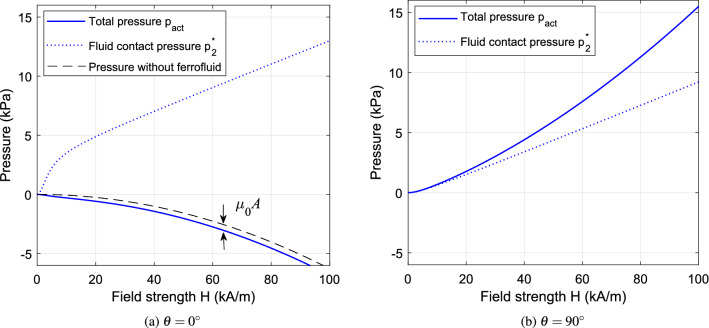
Dependency of transmitted pressure on field strength, accounting for magnetization saturation: (**a**) With axial field direction; (**b**) With orthogonal field direction.

### Effect of magnetization saturation

To determine the pressure that will arise with high values of field strength ($H\gtrsim H_{0})$, the nonlinear magnetization relation Eq. (6) can be substituted into Eq. (17). Results are shown in Fig. 4 for a ferrofluid having the magnetization function shown in Fig. 5, with saturation magnetization $M_{s}=79$ $\mathrm {kA/m}$ and low-field susceptibility $\chi =18.6$. In the low-field regime, the presence of the ferrofluid changes the total pressure by a factor $\mu _{r}=\chi +1=19.6$ when compared to the case with nonmagnetic fluid, in accordance with Eq. (18). In the high-field regime, the curves deviate from this quadratic relation due to the effect of saturation, and the spread of pressures obtained by changing the field angle is shifted in a positive direction.

For high field strengths such that $M\rightarrow M_{s}$, the effect of saturation on actuation pressure can be analyzed for the two extremum cases with $\theta =0^\circ$ and $\theta = 90^\circ$. For large *H*, the area between the magnetization curve and the line $M=M_{s}$ tends to a constant: $HM_{s}-\int _{0}^{H}M.dH\rightarrow A$, as shown in Fig. 5. It then follows from Eq. (17) that, for the axial field case ($\theta =0^\circ$),

19 $$\begin{aligned} p_{act}\rightarrow -\tfrac{1}{2}\mu _{0}H^{2}-\mu _{0}A \end{aligned}$$

Hence, the (negative) actuation pressure becomes similar to the case with nonmagnetic fluid or air, as shown in Fig. 6a.

The pressure behaviour for the orthogonal field case is shown in Fig. 6b. For this case with $\theta =90^\circ$, $H_{n}=0$ and the limiting behaviour from Eq. (17) is

20 $$\begin{aligned} p_{act}\rightarrow \tfrac{1}{2}\mu _{0}H^{2}+\mu _{0}HM_{s}-\mu _{0}A \end{aligned}$$

In this case, there is still a significant contribution to the overall pressure from the fluid magnetization, even when $H>M_{s}$. Nonetheless, for very high strength fields $H\gg M_{s}$, we obtain $p_{act}\rightarrow \frac{1}{2}\mu _{0}H^{2}$ and so the total pressure becomes similar to the case without fluid. It can also be seen that, for a field strength of 100 kA/m, the orthogonal field generates over twice the magnitude of pressure compared with the axial field (15.49 kPa compared with 6.83 kPa). Clearly, the symmetry of force generation seen in the linear case, where the pressure magnitude $|p_{act}|$ is the same for $\theta =0^{\circ }$ and $\theta =90^{\circ }$, is not preserved for large fields.

## Example cases

In this section, two basic linear actuator systems with realistic geometries are examined. The chosen cases can be analyzed using relatively simple magnetic circuit equations.

### Case 1: Axial-field mode of operation

**Figure 7:**
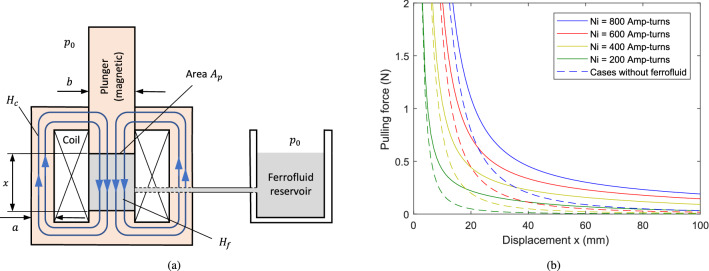
Example actuator system with axial-field mode operation (Case 1): (**a**) schematic; (**b**) force behaviour.

Consider a solenoid-type actuator shown in Fig. 7. In practice, this type of actuator can be made axisymmetric, where the stator iron forms a closed cylinder with a central iron plunger, around which the coil is wound. The ferrofluid that fills the central chamber must flow freely in and out of the chamber, e.g. via a reservoir at ambient pressure. For this study, a planar construction with constant out-of-plane depth *d* is considered so that uniform flux density within the iron core segments and ferrofluid can be reasonably assumed. Given that the coil has *N* turns with current *i*, applying Ampere’s magnetic circuit law yields

21 $$\begin{aligned} H_{c}(l_{c}-x)+H_{f}x=Ni \end{aligned}$$

where $H_{c}$ and $H_{f}$ are the field strengths within the iron core and ferrofluid-filled central chamber, respectively. The displacement of the plunger is denoted by *x*, and $l_{c}$ is the flux path length through the iron when $x=0$. Continuity of $B_{n}$ at the interface between the ferrofluid and plunger implies $B_{c}=B_{f}$ and so

22 $$\begin{aligned} H_{c}+M_{c}(H_{c})=H_{f}+M_{f}(H_{f}) \end{aligned}$$

For given values of *x* and *i*, Eqs. (21) and (22) can be solved numerically to determine $H_{c}$ and $H_{f}$ based on known magnetization functions for the core material and ferrofluid. As magnetic saturation will occur within the iron core at much higher flux densities than the ferrofluid, a constant permeability $\mu _{rc}\gg \chi _{f}+1$ may be adopted for the core material. Then, $H_{c}$ can be eliminated from Eqs. (21) and (22) to obtain

23 $$\begin{aligned} H_{f}=\frac{\mu _{rc}Ni}{\left( 1+\chi _{f}(H_{f})\right) \left( l_{c}-x\right) +\mu _{rc}x} \end{aligned}$$

where $\chi _{f}(H_{f})=M_{f}(H_{f})/H_{f}$ is the (nonlinear) susceptibility of the ferrofluid. This equation can be solved using an iterative approach where an initial value of $\chi _{f}$ is used to calculate $H_{f}$ and then the value of $\chi _{f}$ updated based on $M_{f}(H_{f})/H_{f}$. This is repeated until convergence.

Assuming the fluid chamber is connected to a reservoir at ambient pressure, and gravitational effects are negligible, the net force on the plunger under static conditions will be $F_{m}=A_{p}(p_{act}-p_{0})$ with $A_{p}=bd$. From Eq. (17), this gives

24 $$\begin{aligned} F_{m}=bd\,\left( p_m(H_{f})-\mu _{0}\tfrac{1}{2}H_{f}^{2}-\mu _{0}H_{f}M_{f}\right) \end{aligned}$$

Force values calculated using Eqs. (23) and (24) are shown in Fig. 7b. These results are for $a=10$ mm, $b=20$ mm, $d=40$ mm, and $l_{c}=20$ mm. The ferrofluid properties correspond to the magnetization curve shown in Fig. 5 and the relative permeability of the core material is taken as $\mu _{rc}=1,000$. For small displacement, very high pulling forces can be produced. However, the force decreases rapidly as the length *x* of the central chamber is increased. The presence of ferrofluid has little effect on the force generation at small displacements because the ferrofluid magnetization is highly saturated and so does not impact significantly on the total field. For larger displacements (and therefore lower forces), the presence of the ferrofluid is seen to increase the force generation significantly when compared with the case without ferrofluid.

### Case 2: Orthogonal-field mode of operation

**Figure 8:**
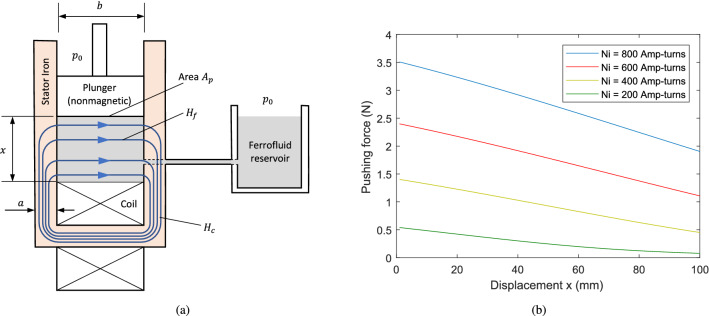
Example actuator system with orthogonal-field mode operation (Case 2): (**a**) schematic; (**b**) force behaviour.

Consider, as an example of orthogonal mode actuation, the system shown in Fig. 8a where the plunger is made of nonmagnetic material and the flux path through the fluid is orthogonal to the actuation axis. As for Case 1, a 2-D planar geometry with depth *d* is assumed. For the flux circuit that passes through the core material and ferrofluid, the following magnetic circuit equation can be applied

25 $$\begin{aligned} H_{c}(l_{c}+x)+H_{f}b=Ni \end{aligned}$$

Here, $l_{c}$ is the path length through the iron when $x=0$. The mean path length of the flux within the iron has been taken as $l_{c}+x$. It is also assumed that there is no flux leakage from the iron core or ferrofluid, and that the flux through the fluid is uniform and orthogonal to the actuation axis. Conservation of the total flux passing through the iron core and fluid implies

26 $$\begin{aligned} a\left( H_{c}+M_{c}(H_{c})\right) =x\left( H_{f}+M_{f}(H_{f})\right) \end{aligned}$$

where *a* is the core width. Introducing the (nonlinear) ferrofluid susceptibility as $\chi _{f}(H_{f})=M_{f}(H_{f})/H_{f}$ and describing the core material magnetization by a constant relative permeability $\mu _{rc}\gg \chi _{f}+1$, gives

27 $$\begin{aligned} H_{f}=\frac{\mu _{rc}Ni}{\left( 1+\chi _{f}(H_{f})\right) \left( l_{c}+x\right) x/a+\mu _{rc}b} \end{aligned}$$

Assuming the fluid chamber is connected to a reservoir at ambient pressure and gravitational effects are negligible, the net force on the plunger under static conditions will be $F_{m}=bd(p_{act}-p_{0})$. Again, the actuation pressure can be evaluated using Eq. (17). However, in this case, $H_{n}=0$ and $H_{t}$ is continuous across the interface so that the resultant force depends only on the magnetization pressure:

28 $$\begin{aligned} F_{m}=bd\,p_m(H_{f}) \end{aligned}$$

Values of this force, calculated using Eqs. (27) and (28), are shown in Fig. 8b for a system where the geometric parameters and ferrofluid properties match the values used in Case 1 ($a=10$ mm, $b=20$ mm, $d=40$ mm, $l_{c}=20$ mm, $M_{s}=79$ $\mathrm {kA/m}$, $\chi _{f}=18.6$, $\mu _{rc}=1,000$). For this design of actuator, the force decreases more slowly as the displacement increases, when compared with Case 1. The maximum force decreases by approximately 40% over a stroke length of 100 mm. This behaviour can be explained by the fact that the field strength within the fluid is less sensitive to the value of *x*, as the term $\mu _{rc}b$ in the denominator of Eq. (27) tends to dominate. However, the downside for this mode of operation is that the peak force for small *x* is greatly reduced in comparison with the axial field actuator case. It may be concluded that, for orthogonal mode operation, high peak force is sacrificed in return for increased force over a large stroke length.

## Experimental and numerical results

### Bilateral actuator design and testing

**Figure 9:**
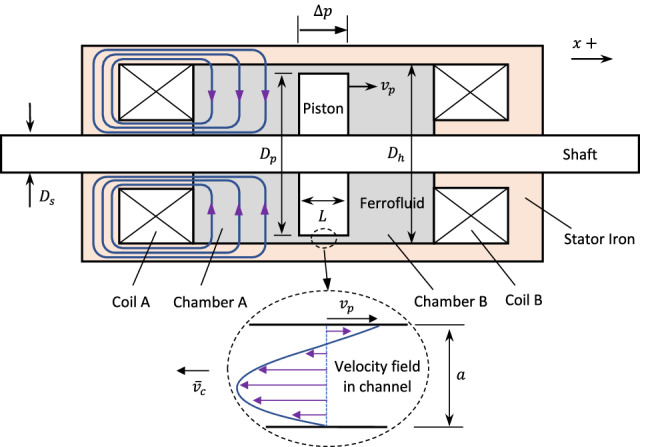
Bidirectional ferrofluid actuator showing magnetic flux lines and fluid flow due to current in coil A.

A novel design of a bidirectional ferrofluid actuator is shown in Fig. 9. This actuator is axisymmetric in form, having a central shaft and piston within the cylindrical bore of a steel tube. The design is based on orthogonal-mode operation where the main mechanism for force generation is the isotropic pressure within the ferrofluid due to the electromagnetic field from the coils. By energizing the coil at one end of the cylinder, the field strength can be increased in the nearest chamber to produce a pressure difference across the piston.

Although the mode of operation is similar to the example system in Case 2, there are some important differences. As the shaft is nonmagnetic, the return path for the flux is through the ferrofluid to a ferrous core within the coil. This results in flux lines that are not entirely parallel to the piston face. Consequently, there is a contribution to actuation pressure from the normal component of the field at the piston surface. For this design, an accurate analytical model of force generation cannot be easily derived. An additional feature of the actuator is that the fluid chamber is sealed and so the fluid must flow around the piston from one chamber to the other. This requires a radial clearance between the piston and cylinder bore, and the size of the clearance will influence the rate of fluid flow. A simple interpretation of the actuation principle is that the magnet field generated at one end of the cylinder will tend to suck the ferrofluid to the same side of the piston, resulting in motion of the piston in the opposite direction, or in the generation of a force if the actuator is blocked.

**Figure 10:**
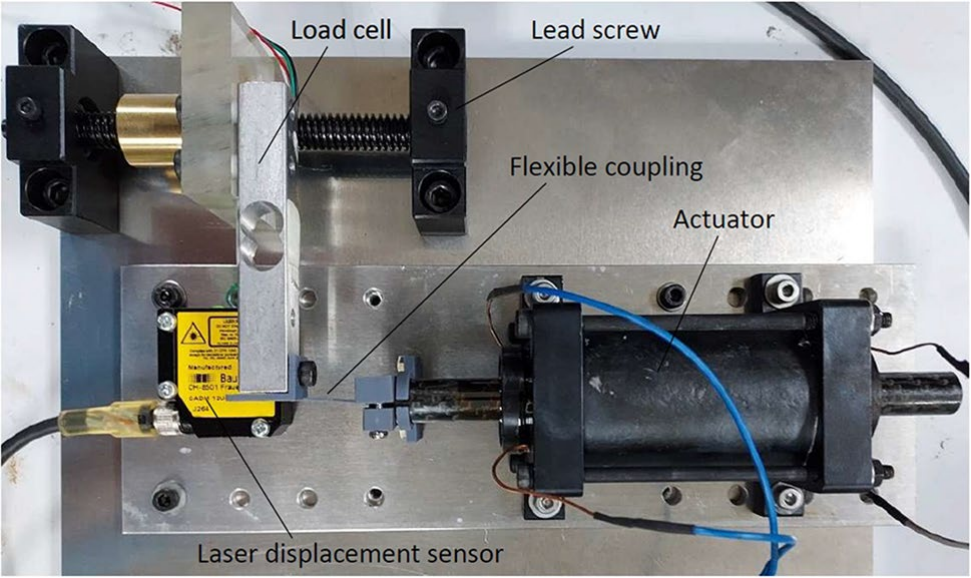
Experimental setup for force and displacement measurements with piston-type ferrofluid actuator.

An experimental setup of this type of actuator with instrumentation for measuring force and displacement is shown in Fig. 10. A load cell is positioned to measure the blocked force from the actuator over a range of shaft displacements. Bearing/seal units are located at each end of the actuator to provide low friction support of the shaft and prevent leakage of the ferrofluid. The total range of motion is approximately 16 mm. Both coils have 155 turns of solid copper wire of size AWG21. The cylinder was filled with Ferrotec EMG901 ferrofluid, which is an oil-based suspension of magnetite particles (11.8 % by volume) having small-field susceptibility $\chi _{f}=7.18$ and saturation magnetization $M_{s}=52.5$ $\mathrm {kA/m}$^25^. Further details are given in Table 1.

**Figure 11:**
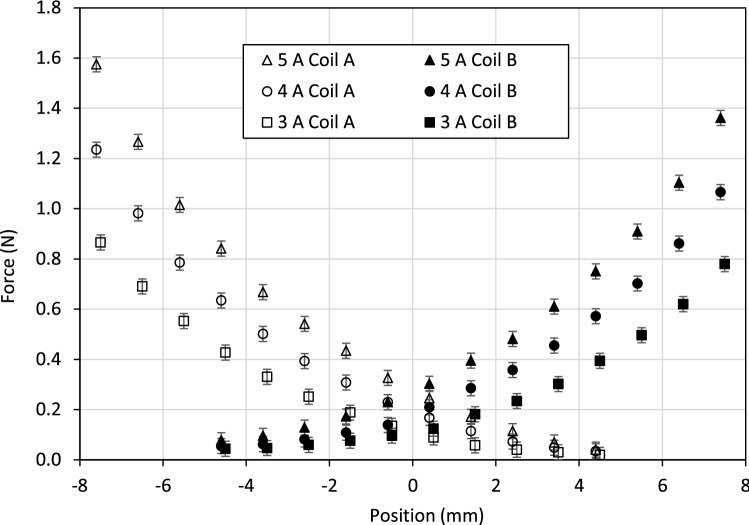
Measured actuation forces for different current values and piston positions. Results are shown for static (blocked) operation with current supplied to a single coil at each end of the actuator.

The measured actuation force is shown in Fig. 11 for selected coil current values. The position variable *x* is the displacement of the piston from its central position, as measured by a laser distance sensor (resolution 20 $\mu$m). Two sets of results are shown in the graph, corresponding to energizing of a single coil at each end of the actuator. The force behaviour is approximately symmetric, as expected from the symmetry of the design. The decrease in force as the distance between the piston and energized coil increases is more rapid than in the example Case 2 (Fig. 8) due to the increasing length of the flux path through the fluid. For this design, the peak force is approximately 1.7 N and the force at the central position is 0.3 N, based on a maximum current of 5 A. Larger current values can be supplied to the coils, as heat dissipation is provided by the surrounding fluid. However, 5 A was deemed suitable for verification of steady-state behaviour without causing significant temperature increases that could affect the ferrofluid magnetization properties.

**Table 1:** Experimental actuator dimensions and properties.

Parameter	Value	Parameter	Calculation	Value
Piston diameter ($D_{p}$)	35 mm	Piston area $(A_{p})$	$\frac{\pi }{4}\left( D_{p}^{2}-D_{s}^{2}\right)$	849 $\mathrm {mm^{2}}$
Housing inner diameter ($D_{h}$)	36 mm	Channel width (*w*)	$\frac{1}{2}\left( D_{h}-D_{p}\right)$	0.5 mm
Shaft diameter ($D_{s}$)	12 mm	Channel length (*l*)	$\frac{\pi }{2}\left( D_{h}+D_{p}\right)$	110 mm
Piston thickness (*L*)	6 mm	Ferrofluid low-field susceptibility $(\chi _{f})$		7.18
Ferrofluid density ($\rho$)	1430 $\mathrm {kg/m^{3}}$	Ferrofluid saturation magnetization $(M_{s})$		52.5 $\mathrm {kA/m}$
Ferrofluid viscosity ($\mu$)	0.008 Pa s	Coil turns (*N*)		155

### Numerical results

**Figure 12:**
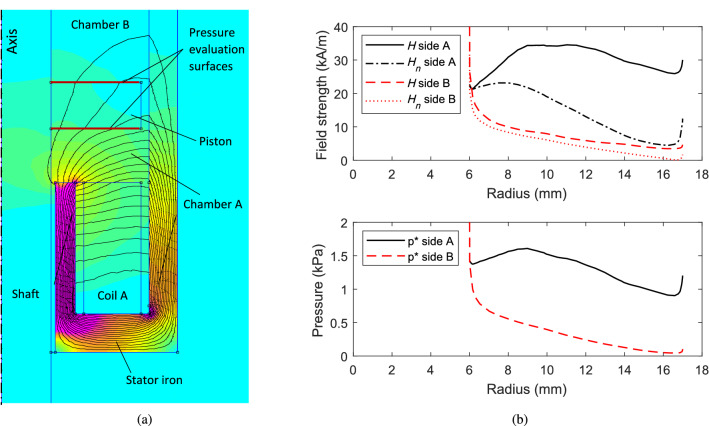
Finite element modelling results with current of 5 A through coil A: (**a**) flux density plot; (**b**) computed field strengths and fluid pressures at piston surfaces.

To predict the force generation characteristics of the tested actuator, a finite element (FE) model was created using FEMM software^26^. Nonlinear magnetization properties of the solid materials and ferrofluid were incorporated based on empirically determined *B*-*H* curves. Illustrative results are shown in Fig. 12. The numerical solution from the FE analysis must be post-processed to determine the pressures and resultant force acting on the piston. As the shaft and piston are nonmagnetic, the resultant force depends only on the fluid pressure on opposing sides of the piston, which can be evaluated from the field vector $\varvec{H}$ and magnetization vector $\varvec{M}$ for the fluid at the interface. To calculate the resultant force, the pressure is integrated over the surface area, according to

29 $$\begin{aligned} F_{m}=2\pi \int _{D_{s}/2}^{D_{p}/2}\left( p_{A}^{*}(r)-p_{B}^{*}(r)\right) r\,dr \end{aligned}$$

where $p_{A,B}^{*}$ are evaluated using Eq. (16). The force is computed by numerical integration of Eq. (29) using data from the FE model, as shown in Fig. 12b. Similar patterns of flux and pressure distributions are obtained for other current values, but scaled in magnitude.

It is clear that large force generation requires a large difference in the field strength on each side of the piston. For this system, the piston thickness is only 6 mm, and so leakage of flux through to the opposite chamber reduces the resultant force to some degree. Increased force could be achieved with a thicker piston, but this would either reduce the stroke length of the actuator, or require an increase in actuator length.

**Figure 13:**
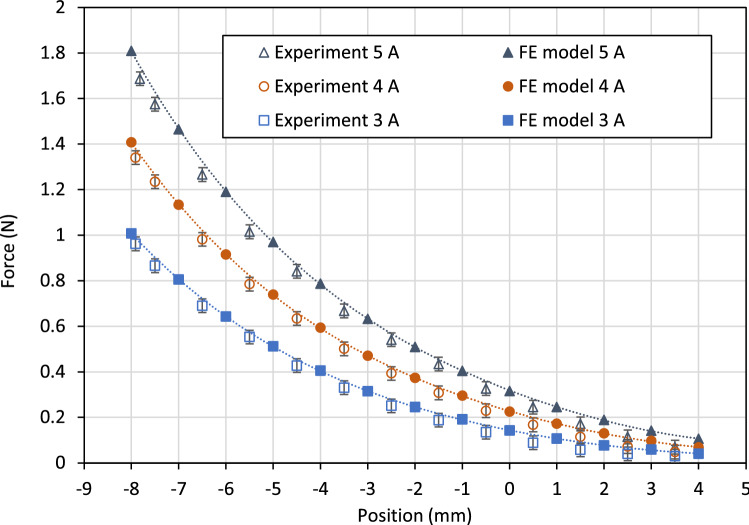
Comparison of experimental and finite element modelling results for energizing a single actuator coil. The static actuation force is shown for different piston positions and coil current values.

Figure 13 shows the results obtained from the FE model, together with equivalent force data from the experimental tests. It can be seen that there is good agreement between the two sets of results, although the experimental force values are consistently lower than the predicted values. In addition to approximation errors in the FE solution, plausible explanations include: 1) air bubbles, or incomplete filling of the fluid chamber causing a reduction in pressure difference across the piston; 2) inaccuracies in the material magnetic models due, for example, to a variation in properties over time or temperature. Nonetheless, the underlying theory and operating principle for the actuator are shown to be appropriate, and therefore provide a foundation for further design explorations and optimization.

### Velocity effects

**Figure 14:**
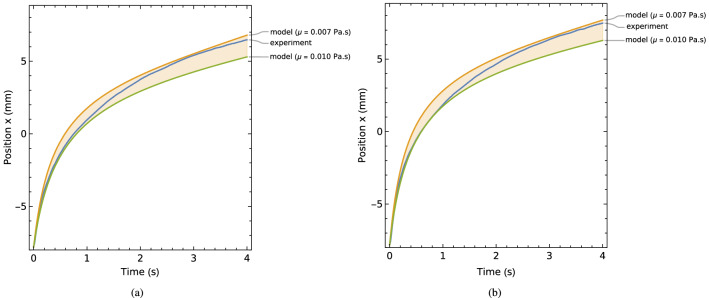
Displacement response of the tested actuator following coil activation with current of (**a**) 4 A and (**b**) 5 A. Results from model-based simulations are also shown for each case.

Under dynamic conditions, the motion response of the actuator is dependent on additional pressure changes associated with fluid flow, as well as any frictional effects arising at the bearings and seals. These effects were quantified experimentally by performing tests where a single actuator coil was energized, driving the piston from one extremum position to the other without any external load or constraint. Results are shown in Fig. 14 for cases with coil currents of 4 A and 5 A. During each test, the piston accelerates rapidly at first but, following a short transient period, the response shifts to quasi-steady-state conditions where the force due to the applied field is balanced by the force due to fluid flow. The actuation force, piston velocity and flow-related force then decrease smoothly as the piston moves further from the energized coil.

To describe the dynamic behaviour of the actuator, the equation of motion for the shaft and piston (having combined mass *m*) may be considered as follows, where $F_{T}$ denotes the transmitted force, $F_{v}$ is the drag force due to fluid flow and $F_{m}$ is the force due to the applied field, as previously defined:

30 $$\begin{aligned} F_{T}=F_{m}(x,i)-m\ddot{x}-F_{v} \end{aligned}$$

To determine the force due to fluid flow, the situation in Fig. 9 is considered, where the piston is moving to the right (positive *x* direction) with a velocity ${\dot{x}}=v_{p}$. The fluid is displaced in the negative *x* direction and flows through the channel formed between the piston and housing. The velocity boundary conditions inside the channel are zero at the housing wall and $v_{p}$ at the piston surface. For the conducted tests, the mean flow velocity satisfies ${\bar{v}}_{c}<0.5$ m/s, and so the flow Reynolds number takes low values ($\textrm{Re}<40$) indicating that the Hagen-Poiseuille model for laminar flow may be applied within the thin annular channel. The velocity profile is parabolic, as shown in Fig. 9, and the volume flow rate *Q* is related to the pressure gradient $\frac{dp}{dx}$ within the channel according to

31 $$\begin{aligned} Q=v_{p}\frac{wl}{2}-\frac{w^{3}l}{12\mu }\frac{dp}{dx} \end{aligned}$$

Here, $\mu$ is the fluid dynamic viscosity and $w=\frac{1}{2}\left( D_{h}-D_{p}\right)$ and $l=\frac{\pi }{2}\left( D_{h}+D_{p}\right)$ are the width and circumferential length of the channel, respectively. Note that the pressure *p* in this equation is the residual (nonmagnetic) pressure and will be summed later with the magnetic pressure component. The volume flow rate must also match the volume swept out by the piston face area $A_{p}$:

32 $$\begin{aligned} Q=-v_{p}A_{p} \end{aligned}$$

Equating these flow rates and substituting $\frac{dp}{dx}=\frac{\triangle p}{L}$ gives:

33 $$\begin{aligned} \triangle p=v_{p}\left( \frac{wl}{2}+A_{p}\right) \frac{12\mu L}{w^{3}l} \end{aligned}$$

An additional pressure drop (opposing motion of the piston) occurs that is associated with fluid entering and leaving the channel. This pressure drop is difficult to predict precisely as it involves nonlinear entrance/exit effects. According to standard models, the total pressure drop may be expressed

34 $$\begin{aligned} \triangle p_{tot}=v_{p}\left( \frac{wl}{2}+A_{p}\right) \frac{12\mu L}{w^{3}l}+K_{1}\rho \frac{v_{p}^{2}}{2}\left( \frac{A_{p}}{wl}\right) ^{2} \end{aligned}$$

where $K_{1}$ is a loss coefficient, which is taken as 1.5 for this geometry. Consequently, the force resisting the piston motion is

35 $$\begin{aligned} F_{v}=\triangle p_{tot}A_{p}=C_{1}v_{p}+C_{2}v_{p}^{2} \end{aligned}$$

where $$C_{1}=\frac{12\mu L}{w^{3}l}\left( \frac{wl}{2}A_{p}+A_{p}^{2}\right)$$ and $$C_{2}=1.5\rho \frac{A_{p}^{3}}{2w^{2}l^{2}}$$.

Considering Eq. (30) for the case when there is no connection to the actuator ($F_{T}=0$), and adopting the fluid force model from Eq. (35), gives

36 $$\begin{aligned} m\ddot{x}=-C_{1}{\dot{x}}-C_{2}{\dot{x}}^{2}+F_{m}(x,i) \end{aligned}$$

The force from the applied field $F_{m}$ can be described empirically by best-fit polynomial curves, as shown in Fig. 13. Using this data in Eq. (36), together with the parameter values given in Table 1 allows the piston position *x*(*t*) to be solved by numerical integration. Wolfram Mathematica was used for this purpose. Results are shown in Fig. 14 and can be directly compared with the experimental response curves.

In the theoretical model the viscosity is assumed to be constant. However, it is also recognized that the effective viscosity of a ferrofluid may increase due to the shearing effect from particles rotating to align their polarization with the imposed field^27,28^. Additional magnetoviscous effects can arise from particle-particle interactions when shearing is perpendicular to the field direction^9,29^. To account for possible viscosity variation, the simulation results shown in Fig. 14 cover a range of dynamic viscosity values with $\mu =[0.007,0.010]$ Pa.s (which encompasses the manufacturer-indicated value of 0.008 Pa.s at $27^{\circ }$C). It can be seen that, for the experimental results, the apparent viscosity is highest during the first second of motion. This is consistent with the aforementioned magnetoviscous effects, as the field strength is highest when when the piston is near to the energized coil. Additional drag effects may arise due to flow within the actuator chambers, and will be most significant when the chamber length is smallest, i.e. near the start and end of the stroke. Overall, the measured response curves fall within the band of simulation curves for both cases with 4 A and 5 A coil currents, confirming the near-to-linear viscous response behaviour of the actuator.

## Conclusions

In this work, the analysis and experimental verification of force generation with magnetic fluids was motivated by the engineering challenge of achieving large force capacity and stroke length in ferrofluid actuation systems. It has been shown how basic theoretical principles may be applied to achieve this aim, leading to the realization of a novel piston-type electro-fluidic actuator having orthogonal mode operation for positive pressure generation. Even though predicted behaviours were confirmed by experiments, challenges still exist in practical usage of such actuators. Many ferrofluids have a volatile base fluid, which will evaporate over time, and therefore must be replenished after extended use. External magnetic fields can have unwanted influence on the fluid pressure, even causing fluid leakage. In such situations, adequate sealing and magnetic shielding must be employed. The stroke length of a ferrofluid actuator is still relatively small for its overall size, especially when compared with actuators based on step-wise motion. Nonetheless, the present study has shown that, for applications where very smooth and precise continuous motion is required, with almost zero friction, ferrofluid can provide a unique and effective solution for mechanism actuation.

## Data Availability

The datasets generated and/or analyzed during the current study are available from the corresponding author on reasonable request.
